# Effect of poly(ethylene-vinyl acetate) pour point depressant on the cold flow properties and crystallization behavior of soybean biodiesel blends fuel

**DOI:** 10.55730/1300-0527.3308

**Published:** 2021-09-23

**Authors:** Jinbao LIU, Chunhua WANG, Qian LIU, Fen ZHANG, Xiaojie LIU, Min SUN

**Affiliations:** School of Health and Social Care, Shanghai Urban Construction Vocational College, Shanghai, China

**Keywords:** Soybean biodiesel, biodiesel-diesel blends, poly(ethylene-vinyl acetate), cold filter plugging point, crystallization behavior

## Abstract

Although biodiesel-diesel blends are being widely used in diesel engines, investigations on its cold flow properties and crystallization behavior are still scarce. In this paper, poly(ethylene–vinyl acetate) (PEVA) pour point depressant and petroleum diesel were worked together to enhance the cold flow properties of soybean biodiesel. PEVA presented a better positive effect in reducing the cold filter plugging point (CFPP) of biodiesel blends. B40 treated with 1% PEVA exerted the best cold flow properties, and its CFPP was decreased by −14 °C. In addition, the crystallization behavior was changed variously. The sizes of crystals were decreased as well as the number of crystals was increased notably.

## 1. Introduction

Along with social and economic speediness development, the need for petroleum diesel fuels increases day by day. Biodiesel is an alternative diesel fuel derived from vegetable oils, animal fats, and other lipids [1,2]. Interest in developing biodiesel as an alternative fuel has grown in recent years because of its renewability and environmental benefits [3,4].

Nevertheless, biodiesel is always limited by its poor low-temperature flow characteristics. The crystallization of biodiesel components at relatively high temperatures during cold seasons causes fuel starvation and operability problems because solidified materials clog fuel lines and filters [5,6]. Several approaches can be used to solve this issue [7–13], such as adding pour point depressants (PPDs), blending with petro-diesel, branched-chain esters, and winterization. Adding PPDs is highly practical, but the available types of biodiesel PPDs are insufficient, the effects are not ideal and the supply cannot meet the demand. Using biodiesel mixed with petroleum diesel and then adding a PPD can greatly enhance its low-temperature flowability. Many studies [14–16] have investigated biodiesel blends. Xue et al. [17] have studied the effect of poly-alpha-olefin (PAO) on the cold flow properties of waste cooking oil biodiesel–diesel blends. PAO has proved to be an effective cold flow improver for waste cooking oil biodiesel blends; and it is more sensitive to B20 (20 vol.% biodiesel+80 vol.% diesel) than to others blends. B20 treated with 400 ppm PAO exhibited the best depression in cold filter plugging point (CFPP) by 9 °C. Ma et al.[18] have added the methacrylate PPD (10–320) into the soybean biodiesel blends to improve the cold flow properties. The constant addition of PPDs resulted in minimal values of CFPP as the proportion of biodiesel varied from 0 vol.% to 100 vol.%. B60 (60 vol.% biodiesel+40 vol.% diesel) with 1% 10–320 PPD produced the most significant reduction in biodiesel blends, and the CFPP reduced by 7 °C. Also, the dispersants were combined with the methacrylate-benzyl methacrylate-Nvinyl-2- pyrrolidone terpolymers (RMC-MB-NVP, R = C_12_, C_14_, C_16_, C_18_) to improve the cold flow properties of waste cooking oil biodiesel blends [19]. Among them, C_16_MC-MB-NVP (5:1:1) combined with the dispersant of fatty alcohol polyoxyethylene ether (FAPE 7) at 4:1 mass ratio showed the best synergistic effect, and the CFPP of B20 decreased by 10 °C at 2000 ppm. However, given that biodiesel compositions vary with the source and the unsatisfying and low depressive effect on the CFPP, studies on the mechanism of PPD-supplemented biodiesel at low temperatures are required because this mechanism remains unclear.

The present work used poly(ethylene–vinyl acetate) (PEVA) as an effective PPD for biodiesel and petroleum diesel blends to investigate the impacts on cold flow properties of soybean biodiesel blends. Differential scanning calorimetry (DSC), polarizing optical microscopy (POM), and low temperature X-ray diffraction (XRD) were used to investigate the performance mechanism of PEVA-supplemented biodiesel to further develop new biodiesel PPDs.

## 2. Experimental

### 2.1. Materials

Diesel fuel untreated with any other PPDs was obtained from Sinopec Group, Shanghai, China. PEVA pour-point depressant was obtained from Rohmax Corporation, Germany. Soybean oil was obtained from a supermarket (Shanghai, China), and the soybean oil biodiesel (SBD) was prepared in our laboratory by transesterification method according to previous literature [17].

### 2.2. Biodiesel compositions measurements

Agilent 7890A-5975c gas chromatography-mass spectrometer (GC-MS) was used to analyze the biodiesel composition [18]. The GC operation conditions were as follows: HP-Innowax quartz capillary column (60 m × 0.25 mm × 0.25 μm); capillary-column temperature initially raised by 10 °C/min from 60 °C to 150 °C, and then raised by 5 °C/min from 150 °C to 230 °C; interface temperature of 250 °C; injector temperature of 250 °C; diffluent ratio of 100:1; high-purity helium carrier gas with a flow rate of 1 mL/min; and injection volume of 0.2 μL. The compositions of the prepared SBD are listed in Table 1.

### 2.3. CFPP measurements

CFPP corresponds to the temperature where wax crystals have agglomerated in sufficient quantity to cause a diesel fuel filter to plug. At present, CFPP is normally used locally to evaluate the cold flow property of diesel. It was determined using a SYP1022–2 multifunctional low-temperature tester (Shanghai Boli Instrument Co., Ltd.) according to ASTM D6371 standard [20]. In addition, other fuel properties, such as cloud point, pour point, flash point, oxidation stability, kinematic viscosity, and acid value, were also determined, and the fuel properties of the diesel and SBD used in this work are shown in Table 2.

### 2.4. Crystal morphology and crystallization behavior measurements

A DSC27HP differential scanning calorimeter (Mettler Corporation, Switzerland) was used to determine the wax precipitation point. The operating conditions are as follows: 8–10 mg samples were put in standard crucibles. Transition temperatures and enthalpies were determined using a computer during the heating cycle at a scanning rate of 5 °C /min and a range of 30 °C −60 °C.

A DM2500P polarizing optical microscope (Leica Microsystems, Wetzlar, Germany) equipped with a Leica DFC420C digital camera was used to determine the low-temperature phase/crystallization behavior of diesel fuel. A sample was dropped onto a slide and then observed at a cooling rate of 0.8 °C/min from 20 °C to −30 °C, and the micrographs were captured in 1°C increment under a magnification of 100× [21].

X’Pert PRD XRD system (PANalytical Corporation, Netherlands) was used to determine the lattice parameter and structure of wax crystals under the following operating conditions: tube voltage of 40 kV, tube current of 40 mA, graphite monochromator, and Cu Kα radiation (λ=1.542 Å) [22].

## 3. Results and discussion

### 3.1. Composition and fuel properties of SBD

The compositions and fuel properties of SBD are shown in Table 1 and Table 2. As shown in Table 1 and Table 2, SBD consists of various FAMEs with different carbon chains and saturated compositions, and the content of unsaturated FAMEs is 81.88% higher than saturated FAMEs (17.41%). All fuel properties of the prepared SBD satisfied the ASTM D6751 standards. However, due to the relatively higher contents and freezing point of saturated FAMEs, the biodiesel and its blends with petro-diesel used in a diesel engine in cold climates were always limited by their weaker cold flow properties.

### 3.2. Impact of PEVA on soybean biodiesel blends

**Figure 1:**
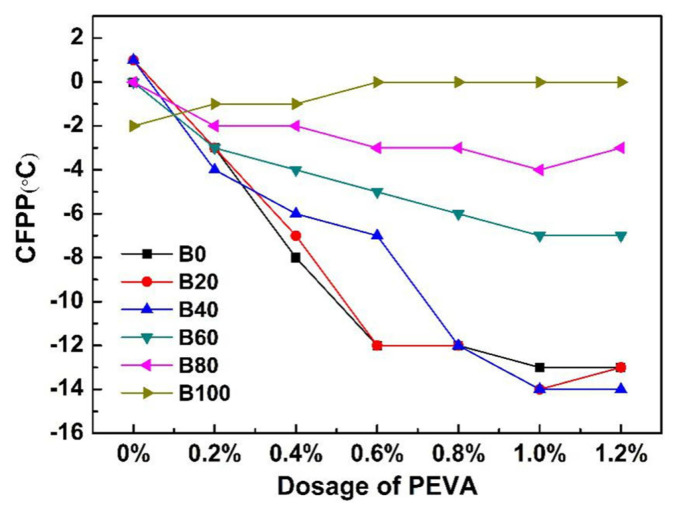
Effect of PEVA on the CFPP of soybean biodiesel blends.

The effect of various dosages of PEVA on the CFPP of soybean biodiesel blends is shown in Figure 1.

As it can be seen from Figure 1, the CFPP of biodiesel blends without PEVA was stable between 0 °C and −2 °C, dosing different PEVA into various biodiesel blends present distinct-different depression effects. PEVA had essentially slight or reactive effects on the CFPP of B100 (pure biodiesel), and the CFPP of B100 was increased from −2 °C to 0 °C after treated with 1% PEVA, indicating that the sensitivity of PEVA to pure SBD was poor. After the biodiesel blends were treated with PEVA, the reduction of CFPP increased firstly and it remained almost unchanged or slightly decreased as the dosage of PEVA above 1 wt.%. The CFPP of B0 (pure diesel), B20 (20vol.% biodiesel + 80vol.% diesel), and B40 (40vol.% biodiesel + 60vol.% diesel) decreased obviously with the PEVA increased from 0.4wt.% to 1.2wt.% than that of B60 (60vol.% biodiesel + 40vol.% diesel), B80 (80vol.% biodiesel + 20vol.% diesel) and B100, and adding 1% PEVA produced the lowest CFPP. Meanwhile, the effects of PEVA on B20 and B40 were similar to those on B0. B0 treated with 1 wt.% PEVA exhibited a relatively low CFPP of −13 °C, while adding 1wt.% PEVA to B20 and B40 produced the lowest CFPP at −14 °C. It indicates that 1wt.% PEVA exhibited a better sensitivity to the biodiesel-diesel blends with a low percentage of biodiesel (B40 and B20). Therefore, given its environmental friendliness and to make full use of more biodiesel, formulated B40 with 1wt.% was considered to be the ideal biodiesel blends with the best cold flow properties.

### 3.3. DSC analysis

**Figure 2:**
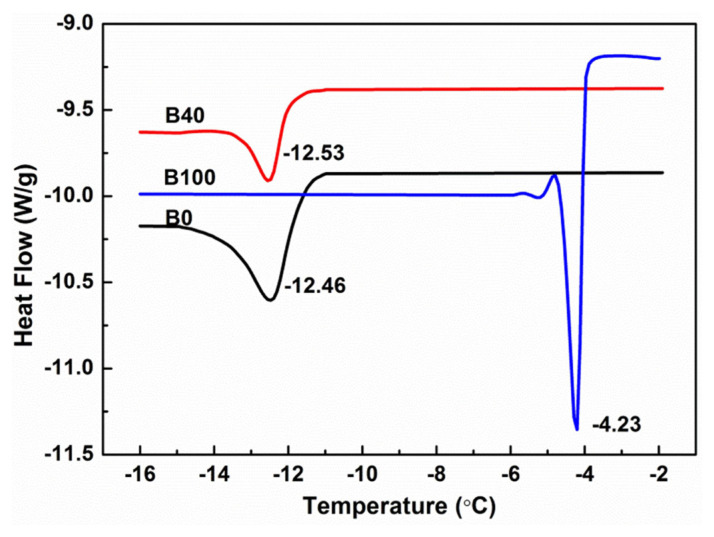
DSC curves of B0, B40, and B100 with 1wt.% PEVA.

DSC can quantitatively analyze energetic changes in the phase-change process in biodiesel blends with PEVA. The starting temperature of peak (onset) in a curve reflects the starting temperature of precipitation of crystals, the slope of peak reflects the rate of precipitation in diesel, and the solid-liquid phase-change energy (ΔH) reflects the stability of the dispersion. The DSC curves and analyses of B0, B40, and B100 with 1% PEVA are shown in Figure 2 and Table 3, respectively.

Table 3 shows that the onset of the peak of B40 was −12.05 °C between B0 (−11.67 °C) and B100 (−3.98 °C). Two crystals precipitated at a low temperature, and peak temperature was consistent with that of onset temperature, indicating the time of three samples from onset to the peak were not very different. The absolute value of ΔH of B40 was the smallest (0.4320J.g^−1^), indicating that the solid-liquid phase-change energy of biodiesel blends was smaller and dispersion was more stable. The crystallization-peak area of B40 was the smallest (3.348), indicating that crystal content was the least. The crystallization-peak slope further revealed that the crystallization rate of B100 was fast, the crystallization rate of B0 was slow, and the crystallization rate of B40 was the slowest. Thus, the CFPP of PEVA-supplemented B40 was lower than that of other biodiesel blends.

### 3.4. POM analysis

**Figure 3:**
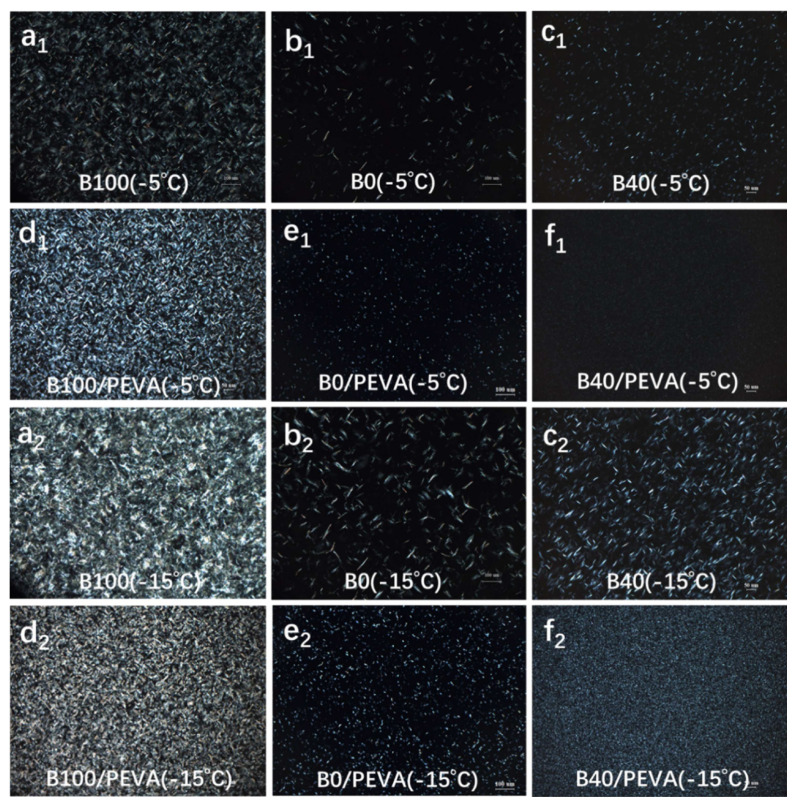
POM images of crystal morphologies of B100(**a_1_** and **a_2_**), B0(**b_1_** and **b_2_**), and B40(**c_1_** and **c_2_**) at −5 °C and −15 °C, 1% PEVA treated B100(**d_1_** and **d_2_**), B0(**e_1_** and **e_2_**), and B40(**f_1_** and **f_2_**) at −5 °C and −15 °C.

POM has proven to be an effective method that can be used to observe changes in wax crystals in the biodiesel blends with and without PEVA [23,24]. The crystal morphology and crystallization behavior of untreated and 1% PEVA treated B0, B40, and B100 were observed by POM at −5 °C and −15 °C, and are shown in Figure 3.

Figure 3 shows dense net-like wax crystals in B100 (Figure 3a_1_), needle-like wax crystals in B0 (Figure 3b_1_), and grain shaped crystals in smaller sizes at −5 °C (Figure 3c_1_). In the presence of PEVA, the crystallization behavior was changed variously. The sizes of crystals were decreased as well as the number of crystals was increased notably (Figure 3dw_1,_ e_1_ and f_1_). The crystals in treated B100 became more regular but intensive-huddled, resulting in the deviation of CFPP (Figure 3d_1_). The shapes of crystals in treated B0 (Figure 3e**_1_**) and B40 (Figure 3f_1_) changed from needle- and grain-like to fine granules, whereas the size of crystals in treated B40 was far less and the quantity was much more than those in treated B0. Following the temperature drops to −15 °C, larger and more crystals were appeared and aggregated in three-dimensional network structures in both untreated and treated B100, B0, and B40, thus losing their flowability at low temperature (Figure 3 a_2~_f_2_). However, two kinds of crystals from biodiesel and diesel precipitated together at low temperatures, one crystal scattered around another crystal and produced some exclusion, thereby inhibiting formation and growth of large and stripe-like crystals by cocrystallization (Figure 3f_1_ and f_2_). Ultimately, the PEVA treated B40 presented a lower CFPP.

### 3.5. XRD analysis

**Figure 4:**
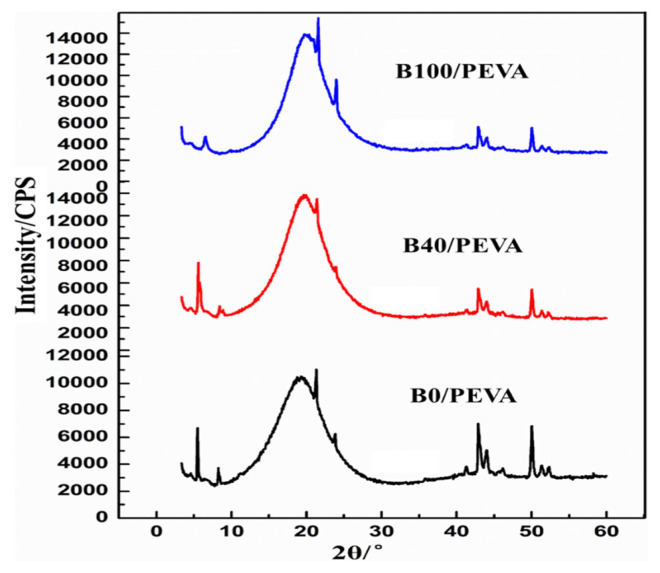
Low-temperature XRD curves of 1wt.% PEVA treated B0, B40, and B100 at −10 °C.

Low-temperature XRD can be used to analyze the lattice parameters and structure of wax crystals in biodiesel [25]. The low-temperature XRD of 1% PEVA treated B0, B40, and B100 at −10 °C are shown in Figure 4.

As it can be seen from Figure 4, the diffusion peak at 10°–30° was quite obvious in B0/PEVA and B100/PEVA. Sharp orthorhombic diffraction peaks were observed at 21.5° and 23.8°, and sharp monoclinic diffraction peaks were observed at 41.3°, 42.9°, 44.0°, 46.2°, 50.0°, 51.3°, and 52.3°, indicating that amorphous and crystalline wax precipitated at low temperatures. The broad of diffusion peaks of B0/PEVA was the first, next was that of B100/PEVA followed by B40/PEVA, so the content of amorphous-wax crystals in pure diesel was more than that in pure biodiesel.

As shown in Table 4 and Table 5, the crystallization-peak areas of three diesels were in the order B0 (136082) > B100 (100045) > B40 (85001), indicating that crystal content in treated B40 decreased at the same temperature. The ratio of orthorhombic to monoclinic peak area in B40/PEVA was 0.14, which is lower than that of 0.15 in B0/PEVA and 0.43 in B100/PEVA. In Table 5, ***A_0_*** refers to the total area of crystallization peak (10.0°–60.0°), ***A****_1_* refers to the total area of sharp monoclinic diffraction peaks ranges from 40.0° to 60.0°, and ***S*** refers to the relative area of orthorhombic area to monoclinic area. This finding indicates that PEVA changed the ratio of two types of crystals by cocrystallization. For another, the ratio of monoclinic peak area (A_1_) to A_0_ in B40/PEVA (0.87) was higher than that of B0/PEVA (0.86) B100/PEVA (0.69). As the aggregation and growth of monoclinic crystals are known to be difficult the crystal size in the PEVA treated B40 was smaller and more easily passed through the filter, resulting in a lower CFPP.

### 3.6. Effect of PEVA on the fuel properties of biodiesel blends

In summary, B40 treated with 1% PEVA presents the best cold flow properties. Table 6 shows the fuel properties of B40, and 1% PEVA treated B40. As it can be seen from Table 6 and Table 2, the fuel properties of B40 are different from that of B0 and B100 due to their huge differences in compositions. After being treated with 1% PEVA, the CFPP of PEVA-supplemented B40 is rand the CP and PP are reduced from 0 °C to −14 °C, and the CP and PP are reduced from 1 °C and −4 °C to −3 °C and −17 °C, respectively. For another, the presence of PEVA in B40 appeared to have no significant effect on other fuel properties of B40, and the flash point, oxidation stability, kinematic viscosity, acid value of B40 just occur a slight increase.

## 4. Conclusions

1. The effects of PEVA on the cold flow properties of soybean biodiesel blends were studied; the CFPP of PEVA-supplemented B40 was lower than that of other blends. B40 treated with 1% PEVA presents the best cold flow properties.
2. Performance mechanisms of PEVA in soybean biodiesel blends are described as follows: PEVA in B40 effectively lowered the crystallization rate of wax crystals, changed the process of crystal growth by cocrystallization, and many small spherical wax crystals existed in B40 that inhibited the formation and growth of the larger crystal, therefore, the CFPP of PEVA-supplemented B40 was lower than that of untreated B40. The crystal size in the PEVA treated B40 was smaller and more easily passed through the filter, resulting in a lower CFPP.

## Figures and Tables

**Table 1:** The compositions of SBD.

Name of fatty acid methyl esters (FAMEs)	Corresponding acid	Mass percent (wt.%)
Methyl hexadecanoate	16:0	11.26
Methyl octadecanoate	18:0	5.34
Methyl oleate	18:1	25.19
Linoleic acid methyl ester	18:2	47.58
Linolenic acid methyl ester	18:3	9.13
Methyl eicosanoate	20:0	0.77
Total saturated FAMEs		17.37
Total unsaturated FAMEs		81.90

**Note:** (16:0) indicates that FAME has 16 of carbons and 0 of unsaturated bonds in the corresponding acid.

**Table 2:** The fuel properties of pure SBD (B100) and pure diesel (B0).

Properties	Test methods	ASTM D6751	SBD	Diesel
Cloud point, °C	ASTM D2500	Report	−1	2
Pour point, °C	ASTM D97	–	−5	−4
Cold filter plugging point, °C	ASTM D6371	–	−2	1
Flash point, °C	ASTM D93	130 min	143	69
Oxidation stability, 110 °C, h	EN14214	3 min	5.2	7.8
Kinematic viscosity, 40 °C, mm_2_/s	ASTM D445	1.9–6.0	4.10	2.47
Acid value, mg KOH/g	ASTM D664	0.50 max	0.38	0.13

**Table 3:** Analysis of DSC of B0, B40, and B100 with 1wt.% PEVA.

Sample	Onset/°C	Peak/°C	ΔH/J.g^−1^	Area
B0	−11.67	−12.46	−1.1491	10.365
B40	−12.05	−12.53	−0.4320	3.348
B100	−3.98	−4.23	−1.0485	8.531

**Table 4:** Analysis of XRD of 1wt.% PEVA treated B0, B40, and B100.

Sample	2–Theta	d(A)	Height	Area
B0/PEVA	21.313	4.1655	2241	14,308
	23.791	3.7368	778	3967
	41.279	2.1853	596	5883
	42.93	2.105	3086	33,243
	44.017	2.0555	1403	15,799
	46.164	1.9648	508	9424
	50.025	1.8218	3641	37,361
	51.343	1.7781	741	8985
	52.301	1.7477	607	7112
B40/PEVA	21.383	4.1519	1694	9482
	23.822	3.7321	173	1406
	41.313	2.1835	327	4029
	42.931	2.1049	1828	20,020
	43.987	2.0568	831	9477
	46.164	1.9648	327	6595
	50.025	1.8218	2341	23,875
	51.346	1.778	522	5883
	52.267	1.7488	382	4234
B40/PEVA	21.575	4.1155	3185	17106
	23.985	23.985	2397	13,069
	41.311	41.311	347	6006
	42.96	42.96	1693	18,053
	43.987	43.987	795	8916
	46.195	46.195	264	5969
	50.026	50.026	2122	21,385
	51.345	51.345	445	5707
	52.333	52.333	334	3834

**Table 5:** Analysis of XRD of 1wt.% PEVA treated B0, B40, and B100.

B0/PEVA			B40/PEVA			B100/PEVA		
S	A_0_	A_1_	S	A_0_	A_1_	S	A_0_	A_1_
0.15	136,082	117,807	0.14	85,001	74,113	0.43	100,045	68,870

**Table 6:** The fuel properties of B40 and 1% PEVA treated B40.

Properties	B40	PEVA treated B40
Cloud point, °C	1	−3
Pour point, °C	−4	−17
Cold filter plugging point, °C	0	−14
Flash point, °C	117	119
Oxidation stability, 110 °C, h	6.1	6.3
Kinematic viscosity, 40 °C, mm2/s	3.16	3.20
Acid value, mg KOH/g	0.21	0.22

## Nomenclature

CFPP: cold filter plugging point
PPD: pour point depressant
PPDs: pour point depressants
SBD: soybean biodiesel
PEVA: poly(ethylene–vinyl acetate)
DSC: differential scanning calorimetry
POM: polarizing optical microscopy
XRD: X-ray diffraction
GC-MS: gas chromatography-mass spectrometer
FAME: fatty acid methyl ester
FAMEs: fatty acid methyl esters
B0: pure diesel
B20: 20vol.% biodiesel + 80vol.% diesel
B40: 40vol.% biodiesel + 60vol.% diesel
B60: 60vol.% biodiesel + 40vol.% diesel
B80: 80vol.% biodiesel + 20vol.% diesel
B100: pure biodiesel
